# Identifying core competencies for practicing public health professionals: results from a Delphi exercise in Uttar Pradesh, India

**DOI:** 10.1186/s12889-020-09711-4

**Published:** 2020-11-17

**Authors:** Sudip Bhandari, Brian Wahl, Sara Bennett, Cyrus Y. Engineer, Pooja Pandey, David H. Peters

**Affiliations:** 1 Department of International Health, Johns Hopkins Bloomberg School of Public Health, Baltimore, MD USA; 2 Indian Administrative Service, Lucknow, Uttar Pradesh India

**Keywords:** Public health competencies, Delphi technique, Human resource development, India

## Abstract

**Background:**

Ensuring the current public health workforce has appropriate competencies to fulfill essential public health functions is challenging in many low- and middle-income countries. The absence of an agreed set of core competencies to provide a basis for developing and assessing knowledge, skills, abilities, and attitudes contributes to this challenge. This study aims to identify the requisite core competencies for practicing health professionals in mid-level supervisory and program management roles to effectively perform their public health responsibilities in the resource-poor setting of Uttar Pradesh (UP), India.

**Methods:**

We used a multi-step, interactive Delphi technique to develop an agreed set of public health competencies. A narrative review of core competency frameworks and key informant interviews with human resources for health experts in India were conducted to prepare an initial list of 40 competency statements in eight domains. We then organized a day-long workshop with 22 Indian public health experts and government officials, who added to and modified the initial list. A revised list of 54 competency statements was rated on a 5-point Likert scale. Aggregate statement scores were shared with the participants, who discussed the findings. Finally, the revised list was returned to participants for an additional round of ratings. The Wilcoxon matched-pairs signed-rank test was used to identify stability between steps, and consensus was defined using the percent agreement criterion.

**Results:**

Stability between the first and second Delphi scoring steps was reached in 46 of the 54 statements. By the end of the second Delphi scoring step, consensus was reached on 48 competency statements across eight domains: public health sciences, assessment and analysis, policy and program management, financial management and budgeting, partnerships and collaboration, social and cultural determinants, communication, and leadership.

**Conclusions:**

This study produced a consensus set of core competencies and domains in public health that can be used to assess competencies of public health professionals and revise or develop new training programs to address desired competencies. Findings can also be used to support workforce development by informing competency-based job descriptions for recruitment and performance management in the Indian context, and potentially can be adapted for use in resource-poor settings globally.

## Background

A competent workforce is a prerequisite for a high performing public health system [1–4]. A strong health system relies on health workers who are competent to provide quality health services that are effective, efficient, integrated, people-centered, safe, and timely [5]. Improving the capabilities of the health workforce—through improvements in their competencies—has the potential to improve health services and health outcomes, and contribute to social and economic development [6]. Alternatively, a lack of health worker competence would contribute to substandard service delivery, harming patients and populations, especially those from vulnerable communities. Substandard quality of care contributes to broader economic and social costs due to disability, lost productivity, and impairment that amounts to trillions of dollars every year [7].

The COVID-19 pandemic has highlighted the importance of core competencies to deliver public health functions, including prevention, detection, and response to disease outbreaks. However, many resource-poor settings struggle to ensure the health workforce has the appropriate public health competencies needed to effectively perform these and other public health functions [8, 9]. In many low-resource settings, funding for training of public health professionals is inadequate; little attention is paid to the health needs of the population during professional training; educational curricula remain outdated; public health training is conducted mostly by medical colleges, which offer narrow perspectives about population-health and restrict access to public health training for non-clinical students; and there is, overall, under-investment and poor governance of the health sector, which may aggravate these challenges [8, 10–13].

Uttar Pradesh is the largest state in India, with almost 230 million people [14]. As with many states in the country and resource-poor settings globally, UP continues to face several health workforce challenges, including a shortage of health workers [15]. Also, there are no requirements for health workers to receive public health training, making it challenging to deliver essential public health functions for population health or professionally manage health services. In addition, there remain challenges related to improving the match between professional competencies and population health priorities, the mix of competencies among the health workforce, and the distribution of professionals across geographical areas—specifically rural and urban areas [16].

The identification of core competencies for public health professionals in UP provides a basis to address some of these challenges. Core competencies are key knowledge, skills, abilities, and attitudes that the health workforce should possess to effectively deliver essential public health functions like epidemiological surveillance, situation assessments, and health promotion [17, 18]. They draw on multiple public health disciplines and are not specific to a single program or topic. Core competencies should be defined for all employees in all positions throughout the public health system. However, competencies may be required at different proficiency levels for different cadres depending on the nature of their job responsibilities. Identification of competencies can help address the mismatch of professional competencies and population health priorities by determining areas where more training, supervision, and support for health professionals is required to meet the health needs of the population. Identification of competencies can also help in developing competency assessment instruments, which can be utilized to identify current competencies in the available health worker workforce and how the competencies are geographically distributed. Policy makers can utilize such information for staff deployment during health emergencies, education and training, and hiring with a view towards fulfilling competency needs [19]. This approach of competency-based human resource planning is particularly important in low-resource settings like UP, where policymakers have to make the optimal use of constrained human resources. This approach allows them to move beyond simply estimating numbers of health workers thought to be needed and instead facilitates planning based on the unique mix of competencies available within the existing health workforce.

Around the world, efforts to develop core competencies for public health professionals have largely been made in High-Income Countries (HICs) and regions [20–22], for the clinical health workforce [23–25], researchers working in resource-poor settings [26], and public health academic programs [27–29]. In India, the Public Health Foundation of India (PHFI), the Union Ministry of Health and Family Welfare (MOHFW), and others have developed core competencies frameworks for Master’s in Public Health (MPH) programs and community medicine fellowships [30–32]. However, competency identification for professionals who are currently practicing public health in low- and middle- income countries (LMICs) is uncommon.

This study builds on these previous efforts. The aim of this study is to develop an agreed list of core competencies for health professionals who have public health responsibilities and are in supervisory or program management roles in UP. Currently, the state has no public health cadre, so these competencies are intended for professionals who have job responsibilities in public health. The identified core competencies are applicable to staff like Medical Officers (MOs), District Program Managers (DPMs), and Additional Chief Medical Officer (ACMO) who are senior to the frontline staff (i.e., ASHAs, Anganwadi workers) and junior to senior management and executive-level staff (i.e., Directors, Additional Directors, Chief Medical Officers) in the UP health system. Examples of responsibilities include developing operational plans to implement national programs, providing assistance in the formulation of village health and sanitation plans, undertaking financial and administrative duties, and organizing in-service training programs for staff in their facilities.

This study was undertaken as a part of broader efforts to strengthen public health worker performance, performance management, and training in the state. Results can be used in UP and adapted for use in other resource-poor settings globally to develop competency-based management systems to aid training, better job analysis, job design, and performance management of human resources for health.

## Methods

We employed the multi-step interactive Delphi technique called Estimate-Talk-Feedback-Estimate (EFTE), a widely-used consensus generating method that solicits opinions of experts through a series of carefully designed questionnaires and face-to-face discussions [33–37].

We used the Delphi technique because of its various advantages including anonymity between participants—which minimizes group discussion biases; iteration with controlled feedback of group opinion—achieved through the use of successive questionnaires allowing participants to amend their views if they want; statistical aggregation of group response—which is shared with the participants, enabling them to see where their opinions lie relative to the group response; and expert input—ensuring that the participants are experts adequately informed in the topic [38].

**Fig. 1:**
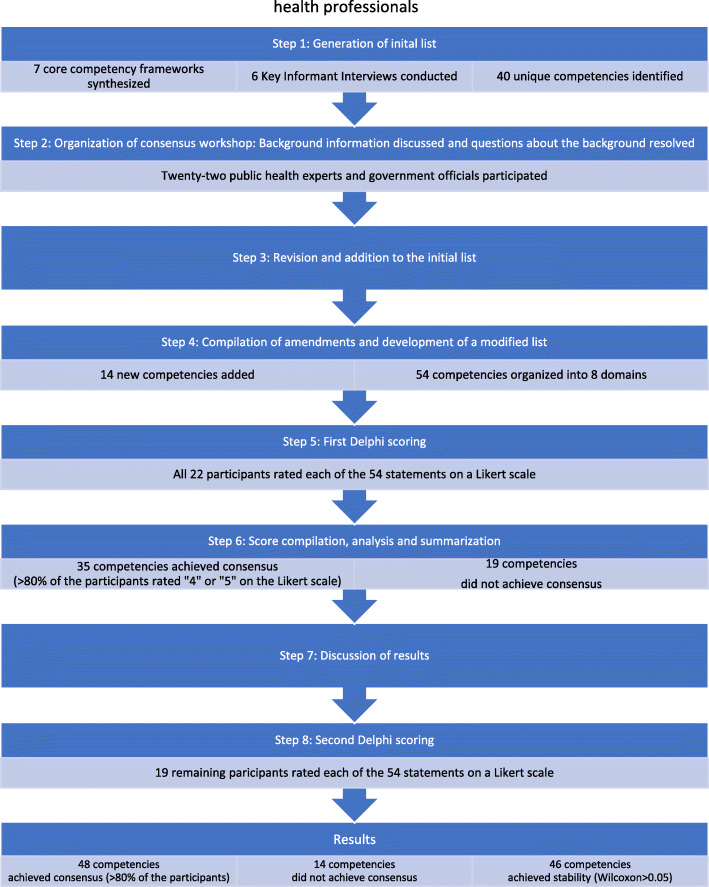
Step diagram of the process and results of identifying core competencies for public health professionals

EFTE is a modified Delphi technique, developed by Nelms and Porter in 1985 to forecast the impact of information technologies on clerical work [34]. Of the many variants of the Delphi technique, we chose to use EFTE because it has a few advantages over other techniques. First, the procedure allows face-to-face interaction and open discussion of ideas. The iterative process of coming to a consensus permits participants to receive and provide feedback in real-time and refine their original positions. Relatedly, many in the health sector may have varied familiarity with the concepts of job competencies as well as public health, so the technique allows participants to more freely exchange information and ideas in real time to have a similar baseline understanding of these concepts. Second, the process works relatively quickly (within a day) and data are obtained immediately. The short turnaround is possible partly because the organizers have more control over participation as the Delphi participants are a “captive audience” working together. In our study, we operationalized the EFTE in the following eight steps (Fig. 1).

1. Generation of an initial list of relevant core competencies

We prepared an initial list of 40 competency statements across eight public health domains after undergoing a narrative review and synthesis of core competency frameworks globally and rapid qualitative interviews with Indian public health experts (see Supplementary Table 2, Additional File 1).

For the narrative review and synthesis, we examined the available core competency frameworks from Canada, Europe, New Zealand, Spain, United Kingdom, United States, and the Americas [21, 22, 39–42]. Competencies listed for Indian MPH curriculums developed by PHFI and the MOHFW were also assessed [30, 31].

Rapid qualitative interviews were conducted with six Indian experts to identify context-specific core competencies to consider. Interviewees with knowledge and expertise in the competency identification of health workers and familiar with the UP context were purposively selected. The interview guide had the following outline (see Additional File 2 for the key-informant interview guide used). In the beginning, we asked respondents about their public health roles and expertise, and their understanding of public health functions and related competencies as it related to the UP setting. This was particularly important as public health tends to hold different meanings in different parts of the world, with some describing it as any health service provided by the government (including government-owned hospitals), while others describe it through the lens of health departments and ministries. We also inquired about the vital public health services that public health managers and supervisors are expected to provide in UP, to clarify the types of public health responsibilities that these health professionals are expected to fulfill, as many cadres in this category do not have clearly defined job descriptions. We explored their understanding of competencies, as this concept can be new to many and the interpretation can be context dependent and focused on what they considered to be the essential competencies that mid-level program managers and supervisors need to possess to deliver public health services in UP. We also asked about the process through which public health professionals acquire these competencies, to understand the current systems and gaps in workforce development efforts. We also asked the interviewees about how they envision addressing some of these gaps they identified to provide insights on how to apply the competency framework locally.

We reviewed the transcripts of the interviews, determined sections relevant to the research question, and inductively developed a coding system. Coding was done independently and manually using the following steps by the first author (SuB). Across the transcripts, the text that addressed each of our questions were highlighted. These highlighted segments were compared and grouped based on similarities. These groupings were then labeled with a code that described the data in them. These steps were iterative in nature, where codes were developed, removed, or split as necessary. Once the coding process was complete, the relationships within the groups and across the groups were explored for any similarities, differences, or contradictions to uncover underlying themes. We then reworked the competency statements to integrate the findings of the interviews.

2. Organization of a consensus workshop

We sought to arrive at a set of public health competencies through a consensus-building process that included a wide range of stakeholders, as this would likely increase the prospects of the results being used in workforce development [43]. A one-day consensus generation workshop was held in Lucknow, Uttar Pradesh, on 26 July 2019. A diverse group of 22 participants with backgrounds in public health, professional education, and human resources for health representing the Government of UP (GOUP), academia, and public health Non-Governmental Organizations (NGOs) in India participated in the meeting. Table 1 provides the demographic characteristics of the participants.

3. Revision and addition to the initial list

**Table 1:** Demographic characteristics of the Delphi participants *(N = 22)*

	Number	Percentage
Gender		
Male	19	86%
Female	3	14%
Current role		
Academic	6	27%
State trainer^a^	4	18%
Senior manager in government	9	41%
Other (e.g., public health NGO)	3	14%
Professional location		
Within Uttar Pradesh	16	72%
Outside of Uttar Pradesh	6	28%

^a^State trainers are the faculty members of the State Institute of Health and Family Welfare. One of their primary responsibilities is to train the newly inducted Medical Officers in the public system on public health topics

The 22 participants were divided into four separate groups, with approximately 6 participants per group. These groups were determined before the workshop to ensure heterogeneity of backgrounds among participants in each group.

A list of the initial 40 draft competencies (prepared in step 1) was given to each participant, who then modified and added to the list individually. Participants were asked to focus on amending the list in light of the critical competencies that they thought were necessary to work successfully as a public health program manager or supervisor in UP. In their groups, participants then discussed major changes and additions to the competency list. However, discussion regarding weights or importance of competency statements was discouraged to avoid groupthink and interfere with the goals of the process.

4. Compilation of amendments, and development of a modified list

The Delphi facilitators in each group (each with graduate training in public health, who were provided training specific to Delphi facilitation) consolidated the written amendments from the participants to decrease duplication and sought clarifications on competency phrasing if required. The facilitators then worked across groups to compile the list of additions and modifications to develop an updated competency list, which included 54 competency statements.

5. First Delphi scoring

This modified list of competency statements was presented to all 22 participants. They individually rated each of the 54 competency statements in terms of their importance on a five-point Likert scale, from 1 as “not at all important” to 5 as “absolutely essential.” Participants then scored each statement on its own merit instead of comparing it against the other proposed competencies. They were encouraged to score based on how important they thought the competency is to provide public health services as a mid-level public health manager in UP currently.

6. Score compilation, analysis, and summarization

The questionnaire results from step 5 were compiled, analyzed, and summarized. The scoring sheets from all the participants were collected and then entered on Microsoft Excel [44]. This compiled data was then analyzed to produce mean, median, mode, range, and percentiles for each competency statement. Results were summarized in a worksheet, which was printed in hard copy and shared with individual groups. The results were also displayed to the panel using a projector. Competency statements were ranked from high (absolutely essential) to low (not at all important) separately with means and medians. These analysis results were used to facilitate the discussion among participants.

7. Discussion of results

Participants discussed the results of at least two domains pre-assigned to their groups. Delphi facilitators guided the discussions, and participants were allowed to question findings and suggest alternatives. Each group then reported back to the plenary on the main points from their discussions.

8. Second Delphi scoring

The same competency list was returned to the 19 remaining participants who were asked to rate these statements again.

### Data analysis

After the workshop, the stability between Delphi scoring steps (steps 5 and 8 listed above) was assessed using Wilcoxon matched-pairs signed-rank test. Previous research shows that it is necessary to ensure that there is enough stability between Delphi scoring rounds to establish that the results are stable and reliable [45]. A statement was considered stable if there was no statistically significant change in responses between the scoring steps for each statement (*p* ≥ 0.05).

Consensus was identified using the percent agreement criterion. A statement was deemed to have reached consensus when over 80% of the participants ranked it as “very important = 4” or “absolutely essential = 5” in the second Delphi scoring step (step 8).

Data from the two Delphi scoring steps were entered into STATA 14.2 and analyzed [46]. For both the scoring steps, the measures of central tendency (mean, median, and mode) and measures of dispersion (range, interquartile range, and standard deviation) were also calculated.

## Results

### Findings from the narrative review: international scenario of core competencies for public health professionals

Based on the narrative review, we found similarities in domains and competencies across various core competency frameworks, as well as differences in their emphasis (see Supplementary Table 1, Additional File). Most of the frameworks highlight the importance of utilizing public health assessment and analysis tools, using communication competencies to improve health outcomes and reduce health inequalities, and translating public health sciences into practice. However, there are also variations and different emphases. For example, New Zealand uniquely specifies competencies related to the knowledge, understanding, and use of culturally appropriate approaches while working with their indigenous population of Maori [42].

In reviewing the similarities and differences across the frameworks, we merged domains and competencies that were similar and selected dissimilar ones that we deemed valuable in the UP context to be considered by the Delphi participants. We also reviewed Indian MPH core competency frameworks that have been proposed by PHFI and MOHFW, which identify 86 and 20 core competencies, respectively.

### Findings from the key informant interviews

Respondents underscored the importance of a variety of competencies, including those related to management. They discussed the significance of financial and human resource management, including the active supervision of teams. Given its importance, we separated management in two domains in the initial competency list—one that focused on policy and program management and the other on financial management and budgeting.

Assessment and analysis skills were also highlighted as important competency areas. Respondents emphasized skills in computing and situation analysis of environmental factors like floods and epidemics, which impact the functioning of the health units. They also discussed communication as another critical competency, which related to the ability of health workers to use interpersonal skills while working with the community and patients. Findings on these areas were incorporated in the initial list of competencies by either expanding on or retaining competency items from the analytical and assessment, and communication domains. The initial list of domains and competencies prepared after the narrative review and key informant interviews is in Supplementary Table 2 in the Additional File.

### Findings from the Delphi workshop

Several changes were proposed in step 3—revision and addition to the initial list. Fourteen new competencies were added. See Table 2 for these additions and amendments, which formed the subsequent competency list that participants rated in the Delphi scoring rounds.

**Table 2:**
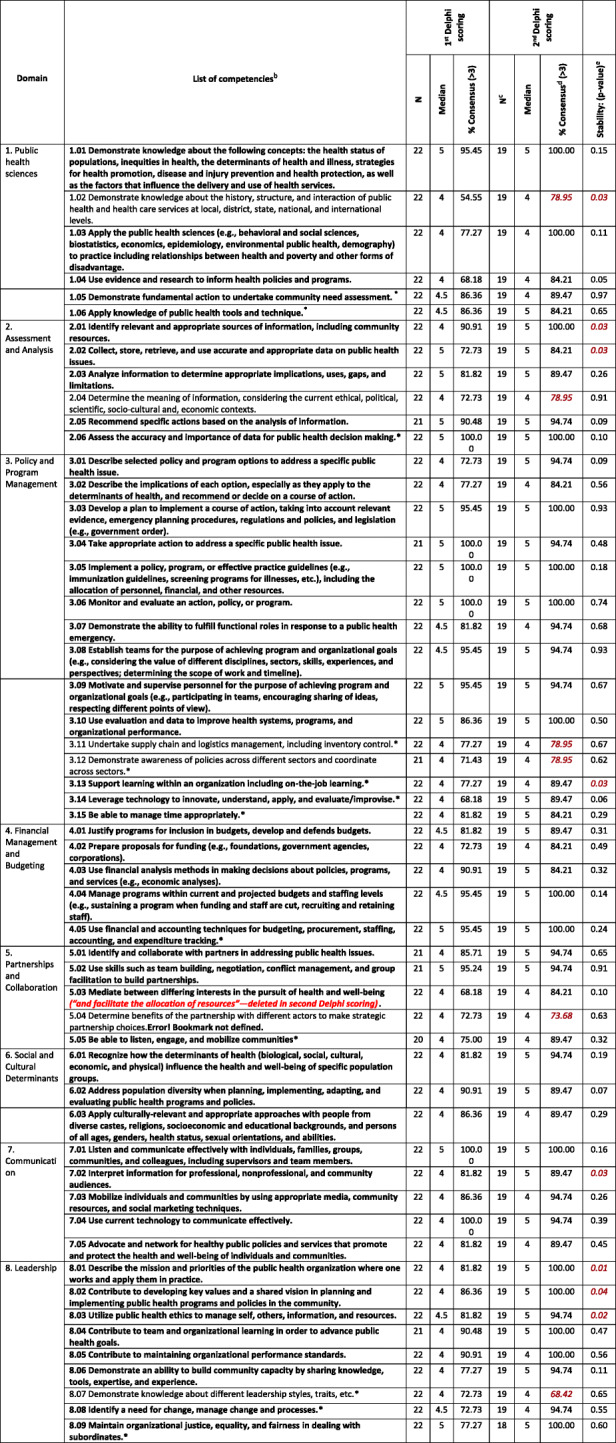
Results from Delphi scoring steps

^*^Competency statement was added in step 3—revision and addition of the list

^b^Bolded statements compose the final list of competencies

^c^Step 8 (second round of Delphi scoring) had three fewer participants compared to Step 5 (first round of scoring). All three participants who left were male, two of whom were government human resource planner, and one was an academic. Note: participants are the same people in each step, as no new participants were added between steps

^d^Consensus was identified using percent agreement criterion. A statement was deemed to have reached consensus when over 80% of the participants ranked it as “very important = 4” or “absolutely essential = 5” in the second Delphi scoring step. The statements where no consensus was reached have been identified in the table above with a red text in the corresponding *p*-value

^e^Stability between Delphi scoring steps was assessed using Wilcoxon matched-pairs signed-rank test. We considered a statement to be stable if there was no statistically significant change in responses between the scoring steps for each statement (*p* ≥ 0.05). Statements where stability was not reached (*p* < 0.05) have also been identified in red text in the table above. Given the importance of these competencies, we left them on our final list. We recognize that subsequent Delphi steps to generate stability in these statements would have been ideal

Public health sciences domain saw two new competencies added—one on the ability to demonstrate action related to community need assessment, and the other on applying knowledge of public health tools and techniques. In the assessment and analysis domain, a competency on the assessment of the accuracy and importance of data for public health decision making was added. The policy and program management domain saw the addition of five new competencies: on the ability to undertake supply chain and logistics management, demonstration of awareness and coordination skills of policies across different sectors, supporting learning within the organization, leveraging technology to innovate and improvise, and the ability to manage time appropriately. The financial management and budgeting domain had one addition related to the use of financial and accounting techniques for budgeting, procurement, staffing, accounting, and expenditure tracking. Participants added two new competencies in the partnerships and collaboration domain—one on determining benefits of the partnership with different actors and another related to being able to listen, engage, and mobilize communities. Participants added three new competencies—knowledge about leadership styles, identifying the need for change and managing such change, and maintaining organizational justice as well as fairness in dealing with subordinates—in the leadership domain.

Stability between the two steps of Delphi scoring was reached in 46 of the 54 statements presented to the participants. Eight statements where stability was not reached (*p* < 0.05) are identified in Table 2 with a red highlight in the corresponding *p*-value. Given their importance —determined by percent agreement criterion—we included seven of them on our final list. The remaining one item did not meet the consensus agreement criterion and was removed from the final list. By the end of the third round, consensus was reached on 48 competency statements across eight domains: (1) public health sciences, (2) assessment and analysis, (3) policy and program management, (4) financial management and budgeting, (5) partnerships and collaboration, (6) social and cultural determinants, (7) communication, and (8) leadership. Six items that did not reach consensus were removed from the final list. Results from the Delphi scoring steps, including the median and proportion consensus for each competency statement, are summarized in Table 2.

## Discussion

We generated expert consensus on 48 core competencies across eight domains for public health professionals assuming mid-level management roles in Uttar Pradesh, India, using an interactive Delphi technique. These competencies represent the current requirements of health professionals to fulfill their job roles and to address the public health needs of UP.

To our knowledge, this is the first attempt to develop core competencies for practicing public health professionals in a resource-poor setting like UP, India. There have been previous efforts in India and other LMICs to generate core competencies in public health education. However, an attempt to identify practice-related competencies is novel. It is helpful to recognize the distinction between core competencies for public health professionals and those for students in educational programs. Educational competencies tend to delineate the skills, knowledge, abilities, and attitudes that students are expected to achieve at the end of their academic programs. They may be organized around traditional academic disciplines like biostatistics, epidemiology, health policy and management, environmental health, and social and behavioral sciences. Professional competencies, on the other hand, reflect the current needs of the workforce, and these are considered to be at the foundation of individual and team success in the workplace [47]. The two are related, and typically the workplace competencies should inform the educational competencies that prepare students for the workplace.

The core competency framework developed in this study covers many of the competencies and domains identified in HICs. However, it also differs in its emphasis on policy and program management, as evident by the number and variety of competencies in this domain. Frameworks from HICs tend to emphasize analysis, assessment, and public health sciences. This difference may reflect the focus of the roles that public health professionals are expected to fulfill in resource-poor settings like UP. Given health systems challenges like lack of access to essential services, overcrowding of clinics, and medicine shortages, there might be an expectation of public health professionals to possess competencies to manage programs in a resource-constrained environment. This distinction may also reflect the weaknesses of health systems in resource-poor settings, and the greater need to train public health professionals in management—a vital lever to strengthening health systems [48].

There were six competencies—which belonged to public health sciences, assessment and analysis, policy and program management, partnerships and collaboration, and leadership domains—that did not achieve consensus in the second Delphi round and were removed from the final list. The removed statements are similar in that they focus on demonstrating knowledge rather than skills and their application, on which many of the statements that achieved consensus focused. For example, statements related to demonstrating knowledge about the history and structure of health services, determining the meaning of information, demonstrating awareness of policies, and demonstrating knowledge about leadership styles did not reach consensus. Such lack of consensus could be attributed to the fact that the final list of competencies is meant for practitioners who are expected to apply competencies in their jobs, rather than possess knowledge alone. On the other hand, the final list of competencies could have risen to the top because they represent the skills, knowledge, abilities, and attitudes necessary or expected of public health professionals in UP.

We identified an important theoretical gap related to the core competency development process, which is relevant for researchers in UP and elsewhere. Core competency frameworks need to be broad enough to be comprehensive but also targeted enough to be relevant for all professionals. However, the public health sector includes many different positions with various responsibilities. The breadth of the public health profession is so wide, that there is confusion as to who should be the target audience for such frameworks. This confusion was described by a recent study by Bornioli, et al. when evaluating the UK’s public health competency framework. As one interviewee in their study aptly asked, “Is [the framework] supposed to cover up to the specialist level? That is a massive breadth of practice. That’s a bit like in education trying to have a curriculum that covers everything from GCSE up to doctorate…” [49].

Some countries have attempted to address this challenge by dividing core competency frameworks for different tiers of public health professionals. For example, the United States has split the core competency framework into three tiers—(1) entry/frontline, (2) program management, and (3) executive/senior management. Such demarcation of public health professionals, however, can be arbitrary and inapplicable to other settings. More important, there is a wide variation of roles within each of these levels that a simple partition is unlikely to solve the need for targeted competencies. Our framework faces a similar challenge—it aims to cover the mid-level professionals which includes public health professionals like medical officers who are usually in charge of primary health care centers at the block level to District Program Managers (DPMs) who work in the district health office and are in charge of public health programs for the entire district.

We propose two major ways to address this issue. The first relates to how we envision the core competency framework to be used. Core competencies are meant to include foundational or crosscutting skills for all individuals working in public health. The onus then falls on the user—policymaker or educator—of the framework to understand the importance of individual core competency to a specific position as they may vary depending on the position. The user should evaluate the types of positions and career trajectories when planning competency-based professional development to ensure that an organization collectively has the strengths across these competencies. So, the framework should not be considered set in stone, but rather a flexible document that end-users can utilize to address their specific needs.

The second way to address this issue could be by identifying functional competencies. While core competencies broadly define the knowledge, skills, abilities, and attitudes for all health professionals regardless of their discipline in a health system, functional competencies are discipline-specific and can build on core competencies. Functional competencies can be developed for groups of professionals like epidemiologists, public health nurses, and public health informaticians.

The development of our framework contributes to the discussions and efforts that have been ongoing in UP, and India more broadly, to build strong public health systems [50, 51]. Specifically, the results of our study contribute to improving public health training, which is timely given the national emphasis on health promotion and prevention through the creation of Health and Wellness Centers (HWCs) under the Ayushman Bharat program [52]. The Indian national health policy (2017) has encouraged states to create a separate public health cadre [53]. As UP contemplates developing its public health cadre, core competencies and subsequent training of current health officials can help address current public health deficiencies. Our framework can also inform the formal training that a public health cadre would need.

This study has two significant limitations. The first limitation relates to the starting point for core competencies *vis-à-vis* essential public health functions or job descriptions. In UP, there is no consensus set of Essential Public Health Functions (EPHFs). Core competencies should map to these EPHFs, which is a set of services that underline the activities that public health workers should perform. In the absence of delineated EPHFs, one could use health workers’ job descriptions as the starting point for core competencies. However, there were challenges associated even with job descriptions. Job descriptions for some mid-level health professionals are either non-existent (e.g., Deputy Chief Medical Officer, district public health nursing officer) or too generic. Also, there might be a high degree of task variation for the same position across the state. So, we depended on the Delphi participants’ expertise to define and interpret health workers’ responsibilities.

The second limitation relates to the use of the Delphi technique, which has numerous variations in how it is operationalized. Such variations have left the technique open to methodological interpretations and criticisms [54, 55]. For this study, the cutoff point of 80%, stability criteria, and the composition of the expert panel were particularly relevant. In terms of the cutoff point, 80% or higher was chosen a priori because this threshold is common in many Delphi studies [56]. However, the theoretical basis for such cutoff is unexplored [54]. In terms of analysis, there were a few statements that did not meet the stability criteria, as indicated in Table 2. It is possible that subsequent Delphi scoring steps may have generated stability in these statements as well.

Regarding the composition of the expert panel, panelists for this study were chosen after extensive consultations and online searches. However, some potential participants declined the invitation due to a lack of availability. These non-participants were similar in their backgrounds from the participants in the Delphi process. The EFTE technique used in this study required face-to-face participation, given the need for real-time discussion and clarification. Conducting the Delphi technique using an online survey may have allowed the inclusion of more stakeholders. Our expert panel also had some limitations in representativeness, as females in the panel were underrepresented, though this reflects the workforce in UP. As such, a different composition of Delphi participants may have resulted in a different final set of competency statements, as experts panels largely dictate the nature and content of the results in Delphi studies [38].

## Conclusions

This article describes the development of core competencies that can be used in a number of specific ways in UP and other resource-poor settings globally, where these competencies may be adapted for local use. First, core competencies can be codified through government orders to help link future efforts in performance management to these competencies. Second, the results of this study can be used to develop a competency assessment instrument. Future research can assess the reliability and validity of that instrument, which can then be used to evaluate levels of competencies of health professionals working in public health management and supervisory roles. The results of the assessment can inform the design of appropriate in-service training programs to address gaps in competencies. Third, these results can be used to evaluate training programs offered through the state and academic institutes to ascertain their ability to meet the competencies expected of public health professionals. Based on the findings of the training evaluations, we can improve training programs by collaborating with relevant stakeholders. This may entail a revision of the training modules through consultation with curriculum designers and trainers, and the development of cadre-specific training modules. Fourth, the findings can be used to map the core competencies against the current job descriptions of various health cadres to identify gaps across domains in knowledge, skills, abilities, and attitudes. The results of the mapping process can be used to amend the job descriptions and make them competency-based. Competency-based job descriptions will assist in recruitment efforts like screening and interviewing, and to define Key Results Areas that enable fair and effective performance management systems. And last, the results provide a unique starting point for the development of a competency-based management system that can be used for workforce planning, recruitment, and development, as well as performance management of public health professionals.

## Supplementary information

**Additional file 1: Supplementary Table 1.** Comparing domains of frameworks of core competencies for public health professionals globally. **Supplementary Table 2.** Initial list of 40 competency statements across eight domains. Supplementary tables to support the conclusions of this article.

**Additional file 2.** Key informant interview guide. Interview guide to support the method of this article.

## Data Availability

The data used and analyzed during the study are available from the corresponding author on reasonable request.

## Abbreviations

ACMO: Additional Chief Medical Officer
COVID: Corona Virus Disease
DPM: District Program Manager
EFTE: Estimate-Feedback-Talk-Estimate
EPHFs: Essential Public Health Functions
GO: Government Order
GoUP: Government of Uttar Pradesh
HIC: High-Income Country
HWC: Health and Wellness Center
LMICs: Low- and Middle- Income Countries
MoHFW: Ministry of Health and Family Welfare
MO: Medical Officer
MPH: Master’s in Public Health
NGO: Non-Governmental Organization
UP: Uttar Pradesh
