# Supplementary material for: Identifying core competencies for practicing public health professionals: results from a Delphi exercise in Uttar Pradesh, India
Source: BMC Public Health. 2020 Nov 17;20:1737. doi: 10.1186/s12889-020-09711-4 (PMC7670983; doi:10.1186/s12889-020-09711-4)
Supplement: Supplementary file 1 — Additional file 1: Supplementary Table 1. Comparing domains of frameworks of core competencies for public health professionals globally. Supplementary Table 2. Initial list of 40 competency statements across eight domains. Supplementary tables to support the conclusions of this article. [file 12889_2020_9711_MOESM1_ESM.docx]

**Supplementary Table 1** Comparing domains of frameworks of core competencies for public health professionals globally

| **Country 🡪** | **Canada (1)** | **Europe (2)** | **New Zealand (3)** | **Americas (4)** | **Spain (5)** | **United Kingdom (6)** | **United States of America (7)** |
| --- | --- | --- | --- | --- | --- | --- | --- |
| **Developed by 🡪** | Public health agency of Canada, Government of Canada | Association of Schools of Public Health in the European Region (ASPHER) | Public Health Association of New Zealand | Pan American Health Organization | Spanish Association of Public Health and Healthcare (SESPAS) and Spanish Society of Epidemiology (SEE) | Public Health England, UK Government | Council of linkages between academia and public health practice |
| Domain #1 | Public health sciences | Methods in public health – quantitative and qualitative methods | Health systems | Health situation analysis | Analyze the health situation of the community | Measure, monitor and report population health and wellbeing; health needs; risks; inequalities; and use of services | Analytical/Assessment Skills |
| Domain #2 | Assessment and analysis | Population health and its social and economic determinants | Public Health Science | Surveillance and control of risks and threats | Describe and analyze the association and impact of risk factors and health problems and the impact of health services | Promote population and community health and wellbeing, addressing the wider determinants of health and health inequalities | Policy Development/Program Planning Skills |
| Domain #3 | Policy and program planning, implementation and evaluation | Population health and its material – physical, radiological, chemical and biological – environmental determinants | Policy, Legislation, and Regulation | Health promotion and social participation | Control diseases and emergency situations | Protect the public from environmental hazards, communicable disease, and other health risks, while addressing inequalities in risk exposure and outcomes | Communication Skills |
| Domain #4 | Partnerships, collaboration, and advocacy | Heath policy; economics; organizational theory, leadership, and management | Research and Evaluation | Policy, planning, regulation and control | Contribute to defining health system management | Work to, and for, the evidence base, conduct research, and provide informed advice | Cultural Competency Skills |
| Domain #5 | Diversity and inclusiveness | Health promotion, health protection, and disease prevention | Community Health Development | Equitable access and quality of individual and public health services | Promote the defense of health in intersectoral policies | Audit, evaluate and re-design services and interventions to improve health outcomes and reduce health inequalities | Community Dimensions of Practice Skills |
| Domain #6 | Communication | Ethics | Te Tiriti o Waitangi | International/global health | Contribute to design and implement health programs and interventions | Work with, and through, policies and strategies to improve health outcomes and reduce health inequalities | Public Health Sciences Skills |
| Domain #7 | Leadership |  | Working Across and Understanding Cultures |  | Encourage social participation and strengthen the degree of control of citizens over their own health | Work collaboratively across agencies and boundaries to improve health outcomes and reduce health inequalities | Financial Planning and Management Skills |
| Domain #8 |  |  | Communication |  | Manage services and programs | Work in a commissioning-based culture to improve health outcomes and reduce health inequalities | Leadership and Systems Thinking Skills |
| Domain #9 |  |  | Leadership, Teamwork, and Professional Liaison |  | Evaluate services and programs | Work within political and democratic systems and with a range of organizational cultures to improve health outcomes and reduce health inequalities |  |
| Domain #10 |  |  | Advocacy |  | Perform sanitary inspections and audits | Provide leadership to drive improvement in health outcomes and the reduction of health inequalities |  |
| Domain #11 |  |  | Professional Development and Self-Management |  | Develop guides and protocols | Communicate with others to improve health outcomes and reduce health inequalities |  |
| Domain #12 |  |  | Planning and Administration |  |  | Design and manage programs and projects to improve health and reduce health inequalities |  |
| Domain #13 |  |  |  |  |  | Prioritize and manage resources at a population/ systems level to achieve equitable health outcomes and return on investment |  |

**Supplementary Table 2** Initial list of 40 competency statements across eight domains

| **Domains and Competency Statements** |
| --- |
| **Public Health Sciences** |
| 1. Demonstrate knowledge about the following concepts: the health status of populations, inequities in health, the determinants of health and illness, strategies for health promotion, disease and injury prevention and health protection, as well as the factors that influence the delivery and use of health services. |
| 2. Demonstrate knowledge about the history, structure and interaction of public health and health care services at local, district, state, national, and international levels. |
| 3. Apply the public health sciences (e.g., behavioral and social sciences, biostatistics, epidemiology, environmental public health, demography) to practice. |
| 4. Use evidence and research to inform health policies and programs. |
| **Assessment and Analysis** |
| 5. Identify relevant and appropriate sources of information, including community resources. |
| 6. Collect, store, retrieve and use accurate and appropriate data on public health issues. |
| 7. Analyze information to determine appropriate implications, uses, gaps and limitations. |
| 8. Determine the meaning of information, considering the current ethical, political, scientific, socio-cultural and economic contexts. |
| 9. Recommend specific actions based on the analysis of information. |
| **Policy and Program Management** |
| 10. Describe selected policy and program options to address a specific public health issue. |
| 11. Describe the implications of each option, especially as they apply to the determinants of health and recommend or decide on a course of action. |
| 12. Develop a plan to implement a course of action taking into account relevant evidence, emergency planning procedures, regulations and policies, and legislation (e.g., government order). |
| 13. Take appropriate action to address a specific public health issue. |
| 14. Implement a policy, program, or effective practice guidelines (e.g., immunization guidelines, screening programs for illnesses, etc.) |
| 15. Evaluate an action, policy or program. |
| 16. Demonstrate the ability to fulfill functional roles in response to a public health emergency. |
| 17. Establishes teams for the purpose of achieving program and organizational goals (e.g., considering the value of different disciplines, sectors, skills, experiences, and perspectives; determining scope of work and timeline). |
| 18. Motivates personnel for the purpose of achieving program and organizational goals (e.g., participating in teams, encouraging sharing of ideas, respecting different points of view). |
| 19. Uses evaluation results to improve program and organizational performance. |
| **Financial Management and Budgeting** |
| 20. Justifies programs for inclusion in budgets, develops and defends budgets. |
| 21. Prepares proposals for funding (e.g., foundations, government agencies, corporations). |
| 22. Uses financial analysis methods in making decisions about policies, programs, and services (e.g., economic analyses). |
| 23. Manages programs within current and projected budgets and staffing levels (e.g., sustaining a program when funding and staff are cut, recruiting and retaining staff). |
| **Partnerships and Collaboration** |
| 24. Identify and collaborate with partners in addressing public health issues. |
| 25. Use skills such as team building, negotiation, conflict management and group facilitation to build partnerships. |
| 26. Mediate between differing interests in the pursuit of health and well-being and facilitate the allocation of resources. |
| **Social and Cultural Determinants** |
| 27. Recognize how the determinants of health (biological, social, cultural, economic and physical) influence the health and well-being of specific population groups. |
| 28. Address population diversity when planning, implementing, adapting and evaluating public health programs and policies. |
| 29. Apply culturally relevant and appropriate approaches with people from diverse castes, religions, socioeconomic and educational backgrounds, and persons of all ages, genders, health status, sexual orientations and abilities. |
| **Communication** |
| 30. Communicate effectively with individuals, families, groups, communities and colleagues. |
| 31. Interpret information for professional, nonprofessional and community audiences. |
| 32. Mobilize individuals and communities by using appropriate media, community resources and social marketing techniques. |
| 33. Use current technology to communicate effectively. |
| 34. Advocate for healthy public policies and services that promote and protect the health and well-being of individuals and communities. |
| **Leadership** |
| 35. Describe the mission and priorities of the public health organization where one works, and apply them in practice. |
| 36. Contribute to developing key values and a shared vision in planning and implementing public health programs and policies in the community. |
| 37. Utilize public health ethics to manage self, others, information and resources. |
| 38. Contribute to team and organizational learning in order to advance public health goals. |
| 39. Contribute to maintaining organizational performance standards. |
| 40. Demonstrate an ability to build community capacity by sharing knowledge, tools, expertise and experience. |

**REFERENCES**

1. Public Health Agency of Canada, Workforce Development Division. Core competencies for public health in Canada [Internet]. Ottawa: Public Health Agency of Canada; 2008. Available from: http://www.phac-aspc.gc.ca/php-psp/ccph-cesp/pdfs/cc-manual-eng090407.pdf

2. ASPHER’s European List of Core Competences for the Public Health Professional [Internet]. 2018 [cited 2019 May 14] p. 1–52. Available from: https://doi.org/10.1177/1403494818797072

3. Public Health Association of New Zealand. Generic Competencies for Public Health in Aotearoa-New Zealand [Internet]. 2007. Available from: https://app.box.com/s/vpwqpz8yyus8d8umucjzbtdi1m111p5u

4. Pan American Health Organization. Core Competencies for Public Health—A Regional Framework for the Americas [Internet]. Available from: https://cursos.campusvirtualsp.org/pluginfile.php/72114/mod_label/intro/competencias-ENG%20final.pdf

5. Benavides FG, Moya C, Segura A, Lluïsa de la Puente M, Portaa M, Amela C. Las competencias profesionales en Salud Pública. Gaceta Sanitaria. 2006 May;20(3):239–43.

6. Public Health England, Public Health Wales, NHS Scotland, Public Health Agency of Northern Ireland. Public Health Skills and Knowledge Framework [Internet]. 2019. Available from: https://assets.publishing.service.gov.uk/government/uploads/system/uploads/attachment_data/file/777278/PHSKF_sub-functions_explained.pdf

7. The Council on Linkages. Core Competencies for Public Health Professionals [Internet]. 2014. Available from: http://www.phf.org/resourcestools/Documents/Core_Competencies_for_Public_Health_Professionals_2014June.pdf
