# Supplementary material for: Identifying core competencies for practicing public health professionals: results from a Delphi exercise in Uttar Pradesh, India
Source: BMC Public Health. 2020 Nov 17;20:1737. doi: 10.1186/s12889-020-09711-4 (PMC7670983; doi:10.1186/s12889-020-09711-4)
Supplement: Supplementary file 2 — Additional file 2. Key informant interview guide. Interview guide to support the method of this article. [file 12889_2020_9711_MOESM2_ESM.docx]

**Key Informant Interview Guide**

Research topic: Core Competencies for Public Health Professionals

Target participants: Human Resource Planner, Manager, Researcher, Educator, and Trainer

Date of interview:

Place of interview:

Time interview started:

Time interview completed:

Name of the interviewer:

Respondent code:

**Questions:**

1. **Current role**
   1. How would you describe your current role as it relates to human resource development?

- (Probes: human resource planner, manager, researcher, educator, trainer)

1. **Understanding of public health**
   1. So, I wanted to understand, from your perspective, what does public health mean?
      - (Probes: population and/or community health, prevention, promotion, treatment, and rehab)
2. **Essential Public Health Services**
   1. What, according to you, are some of the vital public health services that mid-level managers in public sector health officials are expected to provide in UP?
      - (Probes: epidemiological surveillance, health promotion, health situation monitoring, and analysis)
3. **Understanding of competencies**
   1. When you hear the word competency, what comes to your mind?
      1. How would you describe it?
         - (Probes:

- workplace competencies in management and leadership, surveillance;
- competency: knowledge, skills, abilities)
  1. Would you be able to provide examples?

1. **Core competencies to deliver EPHSs**
   1. In your view, what are some of the core competencies that mid-level program management or supervisory level health officials need to have to deliver public health services?
      - - (Probe: mid-level health managers include Medical Officers, Medical Officers In-Charge, Additional Chief Medical Officer, Block Program Manager, Block Community Process Manager, District Program Manager, District Community Process Manager)
        - (Probes:

- Do you think competencies like this… are helpful?
  community health assessments, disease surveillance financial planning and management, communication, policy development and program planning, analytical and assessment.)
  - 1. Can you think of any examples?
  1. Which ones, do you think, are the most critical to do work in an efficient way?
- Probe (Which competencies do you think makes a difference?)
  - 1. What do you think are some of the critical competencies that need to be strengthened?
  1. How often do health officials possess these core competencies?

1. **Current status, challenges, and opportunities in the integration of core competencies**
   1. What do you think are some of the ways they go about getting these core competencies?
      - (Probes: through training, learning from others in the job, learning by doing/experience on the job)
   2. In your knowledge, are there any government plans and programs in which these core competencies—the way you have described them—are being incorporated into workforce development efforts?
      - - If yes, how are these being integrated?
        - (Probes: through training, government orders)
   3. Are there any difficulties in terms of incorporating public health core competencies within the workforce development efforts?
      - - If yes, can you describe what some of those challenges are?
        - (Probes: right kinds of people to do the work, low in the list of priorities, lack of an agreed-upon set of core public health competencies, funds for recruiting people with the required skillset)
   4. Do you think there are any opportunities to incorporate public health competencies in workforce development plans and programs?
      - - If yes, what are those opportunities?
        - (Probes: incorporation in health policy, government orders, training programs through SIHFW)
2. **Cross-cutting**
   1. Since you are the leader in human resource development, and someone who thinks progressively about these issues, if you were to design programs to strengthen core competencies for the future generation, what are some of the key dimensions that you think need to be included in such programs?
      - (Probe: Training needs to be strengthened)
      1. What would you strengthen in the current system of workforce development?
      2. What would you keep the same?
      3. How would you overcome existing challenges, if there are any?
3. **Final remarks**
   1. Is there any additional information that you want to share with us, or any questions that you wish we had asked you?
   2. Do you have any questions for us?
   3. Is there anything else we should ask other health professionals?
